# BMP7 dose‐dependently stimulates proliferation and cadherin‐11 expression via ERK and p38 in a murine metanephric mesenchymal cell line

**DOI:** 10.14814/phy2.13378

**Published:** 2017-09-04

**Authors:** Midori Awazu, Michio Nagata, Mariko Hida

**Affiliations:** ^1^ Department of Pediatrics Keio University School of Medicine Tokyo Japan; ^2^ Kidney and Vascular Pathology University of Tsukuba Ibaraki Japan

**Keywords:** Bone morphogenetic protein 7, cap mesenchyme, development, kidney, mitogen‐activated protein kinase

## Abstract

BMP7 is expressed in ureteric buds and cap mesenchyme of the fetal kidney, mediating branching morphogenesis and survival and priming of metanephric mesenchyme. Although dose‐dependent effects of BMP7 in collecting duct cells have been reported, studies in metanephric mesenchymal cells are lacking. We examined the effects of BMP7 on MAP kinase activation, proliferation, and expression of cadherins in a metanephric mesenchymal cell line MS7 by thymidine incorporation, immunoblot analysis, and quantitative real‐time PCR. The levels of phosphorylated ERK (P‐ERK) and phosphorylated p38 (P‐p38) were not altered at 10 min, 1 h, and 6 h with low‐dose BMP7 (0.25 nmol/L), but were increased at 24 h. At 24 h, P‐ERK was increased with low‐dose BMP7, but not by intermediate‐ (1 nmol/L) or high‐dose (10 nmol/L) BMP7, whereas p38 was activated by intermediate‐dose BMP7. Cell proliferation of MS7 was significantly increased by low‐ and intermediate‐dose BMP7 and decreased by high‐dose BMP7. A p38 inhibitor SB203580 5 *μ*mol/L or a MEK inhibitor PD98059 5 *μ*mol/L abolished BMP7‐stimulated proliferation. Expression of cadherin‐11, an adhesion molecule known to promote cell migration and compaction, was upregulated by intermediate‐dose BMP7. BMP7‐induced cadherin‐11 expression was inhibited by cotreatment with SB203580 and PD98059. Finally, in metanephroi cultured with siRNA for cadherin‐11, the number and thickness of cap mesenchyme were reduced. In conclusion, BMP7 exerts differential effects depending on the concentration; it may expand mesenchymal cells in the stroma where BMP7 concentration is low and may upregulate cadherin‐11 promoting condensation around the tip of ureteric buds.

## Introduction

BMP7 (bone morphogenetic protein 7) is a morphogen expressed in ureteric buds and cap mesenchyme mediating branching morphogenesis and survival/priming of metanephric mesenchyme. Morphogens are diffusible factors that influence developmental patterns by forming gradients, which specify different fates for cells (Bier and De Robertis 2015). Thus, BMPs exert their actions in a dose‐dependent fashion. While dose‐dependent effects of BMP7 in collecting duct cells have been reported, studies in metanephric mesenchymal cells are lacking (Piscione et al. 2001).

BMP7 is one of the major BMPs important in kidney development. BMP7‐deficient kidneys are small and have disorganized architecture characterized by apoptosis of metanephric mesenchyme (Dudley and Robertson 1997). These features are similar to metanephroi cultured with an inhibitor of p38 mitogen‐activated protein kinase (p38) as previously reported (Hida et al. 2002). BMP7 has been reported to be necessary for the maintenance of nephron progenitor cells through another member of MAP kinase c‐Jun N‐terminal kinase (JNK) (Blank et al. 2009). Both JNK and p38 are activated by BMP7 via TAK1. Extracellular signal‐regulated kinase (ERK) of MAP kinase is also shown to be activated by BMP7, although the signaling pathways are not well defined (Grijelmo et al. 2007). We previously showed that blockade of ERK activation inhibited nephrogenesis in a similar manner to p38 inhibition, although the extent was less and the metanephros size was not affected (Hida et al. 2002).

Recently, it has been shown that BMP7 primes nephron progenitors for differentiation (Brown et al. 2013). Thus, BMP7 promotes transition of Cited1+ nephron progenitor cells to Six2‐expressing cells that are inducible by WNT/*β*‐catenin signaling. This action of BMP7 is reported to be mediated by Smad pathway (Brown et al. 2013).

Adhesion molecule cadherins are important in the development stimulating cell migration, compaction, and establishing polarity. Metanephric mesenchyme expresses cadherin 11 (CDH11), which is downregulated during epithelialization. R‐cadherin and cadherin 6 becomes expressed coinciding with the formation of the renal vesicle. E‐cadherin is not seen during the pretubular aggregate stage (Marciano 2016). BMP7 is reported to upregulate or induce E‐cadherin in renal fibroblasts or cultured metanephric mesenchymal cells, respectively (Zeisberg et al. 2005; Gai et al. 2009), but its effect on other cadherins in the kidney is unknown.

In the present study, we investigated the dose‐dependent effects of BMP7 on proliferation, MAP kinase activation, and the expression of cadherins in a metanephric mesenchymal cell line.

## Materials and Methods

### Reagents

BMP7 was from R&D systems (Minneapolis, MI). Antibodies against phosphorylated p38 (P‐p38) and phosphorylated ERK (P‐ERK) were from Cell Signaling Technology (Beverly, MA). Anti‐p38 antibody (C‐20) was from Santa Cruz Biotechnology (Santa Cruz, CA). Anti‐ERK antibody (Erk1/2‐CT, rabbit polyclonal IgG) was from Upstate Biotechnology (Lake Placid, NY). Mouse and rabbit anti‐cadherin‐11 antibodies were from Zymed Laboratories (South San Francisco, CA). Anti‐uvomorulin/E‐cadherin antibody was from Sigma (Saint Louis, MO), and anti‐K‐cadherin antibody (cadherin 6, sc‐31024, sc‐59974) was from Santa Cruz Biotechnology (Santa Cruz, CA). Horseradish peroxidase‐conjugated anti‐mouse IgG and anti‐rabbit IgG were from Amersham (Buckinghamshire, UK). A p38 inhibitor SB203580 and a MEK inhibitor PD98059 were from Carbiochem‐Novabiochem (La Jolla, CA). DMEM, FBS, penicillin, streptomycin, Hanks' balanced salt solution, and trypsin‐EDTA were from Gibco Laboratories (Grand Island, NY).

### Cell line

An immortalized metanephric mesenchymal cell line MS7 was generated from the metanephroi of embryonic day 11.5 homozygous mouse transgenic for H‐2Kb‐tsA (Takemura et al. 2001). MS7 is positive for vimentin and negative for cytokeratin and DBA. Cells were grown in DMEM containing 10% FBS, penicillin 100 U/mL, and streptomycin 100 mg/mL. The cultures were maintained at 37°C in a humidified atmosphere of 95% O_2_–5% CO_2_. Cells were grown to ≈80% confluence and serum deprived for 48 h. Cells were then treated with BMP7, SB203580, PD98059, or vehicle for various times.

### [^3^H]‐Thymidine incorporation

Cells were grown in a 24‐well dish and serum deprived for 48 h. Cells were then treated with low‐ (0.25 nmol/L), intermediate‐ (1 nmol/L), or high‐dose (10 nmol/L) BMP7 or vehicle. In other experiments, cells were treated with 5 *μ*mol/L SB203580 or 5 *μ*mol/L PD98059 in addition to 1 nmol/L BMP7. Cells were then pulsed with 1 *μ*Ci [^3^H]‐thymidine. After incubation for 18 h, cells were washed with ice‐cold PBS and 5% trichloroacetic acid, solubilized in 0.5 N NaOH, and counted by a liquid scintillation counter.

### Quantitative real‐time PCR (qPCR)

Total RNA was extracted from the cells using TRIzol (Invitrogen, Carlsbad, CA) and reverse transcribed into cDNA. qPCR was performed using SYBER Premix Ex Taq II (Takara Bio, Shiga, Japan). The primers used are shown in Table 1.

**Table 1:** Sequences of oligonucleotide primers used in this study

Gene	Forward primer	Reverse primer
*Cdh6*	AAGTTCTCGACGTCAACGACAATG	ACAGCACGCAGGGTCTGAATC
*Cdh11*	CCAATCAGATGGTGGAGCA	GTTGGTCACCTCCGCAGTCA
*Gapdh*	TGTGTCCGTCGTGGATCTGA	TTGCTGTTGAAGTCGCAGGAG

### Immunoblot analysis

Cells were lysed in solubilization buffer containing 20 mmol/L HEPES (pH 7.2), 1% Triton X‐100, 10% glycerol, 20 mmol/L sodium fluoride, 1 mmol/L sodium orthovanadate, 1 mmol/L phenylmethanesulfonyl fluoride, 10 *μ*g/mL aprotinin, and 10 *μ*g/mL leupeptin. Insoluble material was removed by centrifugation (10,500*g*, 10 min). Lysates were resolved by SDS‐PAGE and transferred to PVDF membranes (Immobilon, Millipore, Bedford, MA). Nonspecific binding sites were blocked in Tris‐buffered saline (TBS) (10 mmol/L Tris・HCl, pH 7.4, 0.15 mol/L NaCl) containing 0.1% Tween 20 and 5% skim milk or BSA overnight at 4°C or for 1 h at 25°C. Antibodies were added to TBS containing 0.1% Tween 20 with 5% BSA and incubated with mixing for 24 h at 4°C. The following dilution of antibodies were used: P‐p38 at 1:2000, P‐ERK at 1:1000, p38 at 1:5000, ERK at 1:1000, CDH11 at 1:125. Bound antibodies were detected using the ECL western blotting system (Amersham, Arlington Heights, IL). All experiments were repeated on at least three separate occasions. Bands were quantitatively analyzed by ImageJ software.

### Organ culture

Approval was obtained from the Animal Experiment Committee of Keio University. Culture of embryonic kidneys was performed using low‐volume system (Sebinger et al. 2010). Embryonic day 13 metanephroi were isolated from ICR mice and cultured in sterilized silicone rings on coverslips with 85 *μ*L culture medium. Transfection of siRNA was performed with Lipofectamine 2000 (Invitrogen, Carlsbad, CA) (Davies and Unbekandt 2012). Sequences are shown in Table 2. Next day, the medium was changed to 10% FBS DMEM and cultured for 2 more days. Metanephroi were then fixed with cold methanol. In other sets of experiments, embryonic day 12 metanephroi were transfected with siRNA for 24 h and further cultured in 10% FBS DMEM for 24 h. Ureteric buds and cap mesenchyme were visualized by incubation with anti‐pancytokeratin antibody (Sigma, St. Louis, MO) and anti‐Six 2 antibody (proteintech, Rosemont, IL), each followed by Cy3‐conjugated anti‐mouse antibody or FITC‐conjugated anti‐rabbit antibody. In some experiments, metanephroi were stained with rabbit anti‐CDH11 and anti‐pancytokeratin antibody. Samples were washed three times with PBT, mounted in SlowFade, and viewed under a confocal imaging system (Leica TCS‐SP5, Leica Microsystems, Tokyo, Japan).

**Table 2:** Sequences of siRNA for cadherin‐11

	Sense	Antisense
siRNA a	CAAUUGAUCGUCAUACUGATT	UCAGUAUGACGAUCAAUUGTT
siRNA b	CAAGUUAUAUCCAUGAAGUTT	ACUUCAUGGAUAUAACUUGTT
Irrelevant siRNA	UCUUAAUCGCGUAUAAGGCTT	GCCUUAUACGCGAUUAAGATT

### Statistical analysis

The results are expressed as mean ± SE. Statistical analysis was performed with ANOVA. Statistical significance was determined as *P *<* *0.05.

## Results

### Dose‐dependent effects of BMP7 on ERK and p38 activation

**Figure 1:**
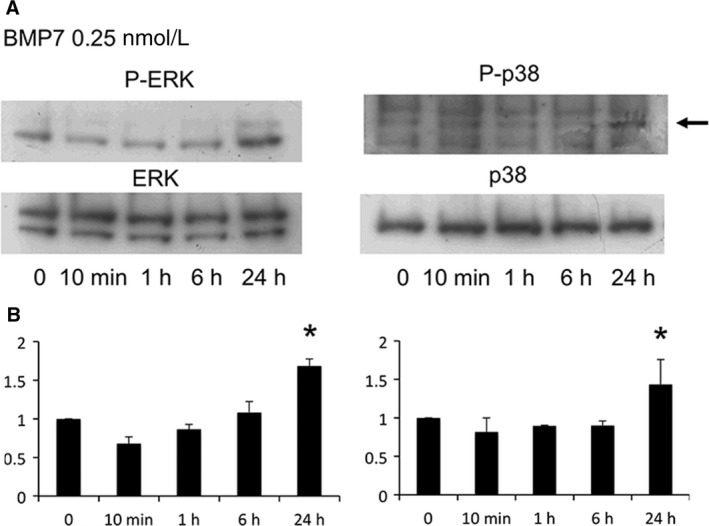
Time course of ERK and p38 activation by BMP7 in metanephric mesenchymal cells. Cells were incubated with low concentration of BMP7 (0.25 nmol/L) for 10 min, 1, 6, and 24 h, and lysed for immunoblot analysis. BMP7 increased phosphorylated ERK (P‐ERK) and phosphorylated p38 (P‐p38) at 24 h. Representative immunoblots (A) and quantitative analysis (B; *n* = 3 for both P‐ERK/ERK and P‐p38/p38). *P < 0.05 versus 0 min.

**Figure 2:**
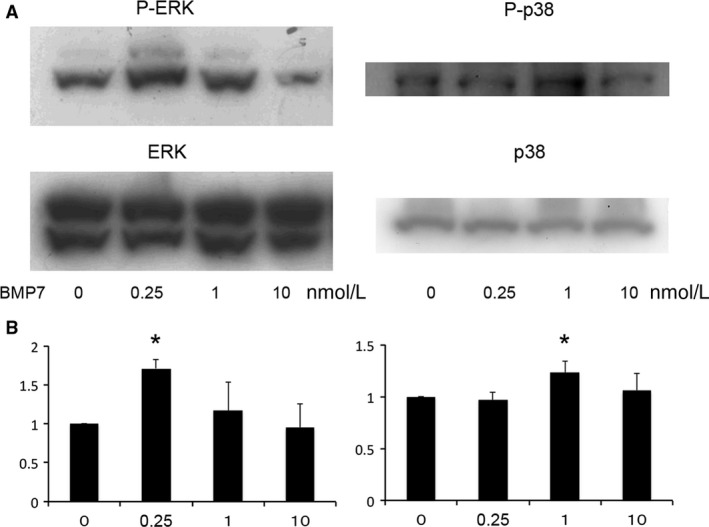
Dose dependency of BMP7 on the activation of ERK and p38 in metanephric mesenchymal cells. Cells were incubated with low‐dose (0.25 nmol/L), intermediated‐dose (1 nmol/L), or high‐dose (10 nmol/L) BMP7 for 24 h, and lysed for immunoblot analysis. Representative immunoblots (A) and quantitative analysis (B; *n* = 5 for P‐ERK and *n* = 6 for P‐p38). *P < 0.05 versus 0 nmol/L.

The level of phosphorylated ERK (P‐ERK) or phosphorylated p38 (P‐p38) in MS7 was not altered by low‐dose BMP7 (0.25 nmol/L) at 10 min, 1 h, and 6 h, but was increased at 24 h (Fig. 1). At 24 h, P‐ERK and P‐p38 were increased by low‐dose and intermediate‐dose (1 nmol/L) BMP7, respectively (Fig. 2).

### Dose‐dependent effects of BMP7 on cell proliferation

**Figure 3:**
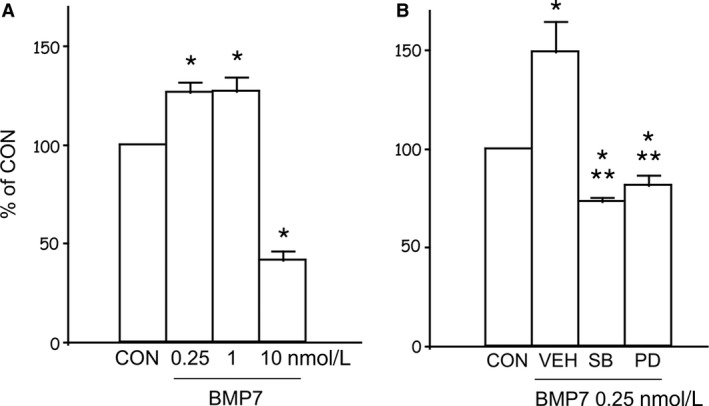
Effects of BMP7 on cell proliferation. (A) Dose dependency. Cells were incubated with various concentrations of BMP7 and [^3^H]‐thymidine for 24 h (*n* = 4). (B) ERK or p38 mediates low‐dose BMP‐induced cell proliferation. Cells were incubated with low‐dose BMP7 with or without PD98059 (PD) 5 *μ*mol/L or SB203580 (SB) 5 *μ*mol/L (*n* = 4). VEH, vehicle; *P < 0.05 versus CON; **P < 0.05 versus VEH.

MS7 cell proliferation assessed by thymidine incorporation was increased by low‐ and intermediate‐dose BMP7, but inhibited by high‐dose (10 nmol/L) BMP7 (Fig. 3A). A p38 inhibitor SB203580 5 *μ*mol/L or a MEK inhibitor PD98059 5 *μ*mol/L abolished low‐dose BMP7‐stimulated proliferation (Fig. 3B).

### Dose‐dependent effects of BMP7 on cadherin‐11 expression

**Figure 4:**
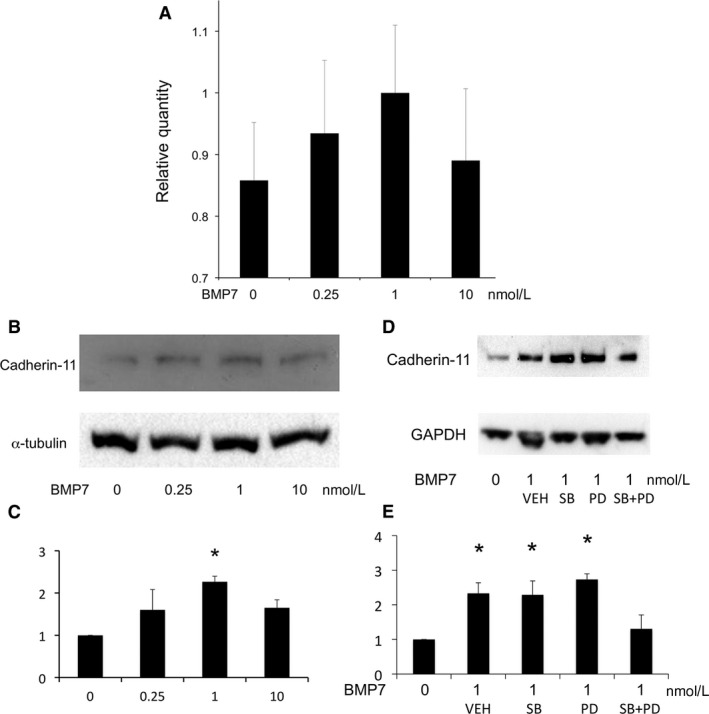
Effect of BMP7 on cadherin‐11 mRNA expression. (A) BMP7 tended to increase cadherin‐11/GAPDH mRNA, assessed by real‐time PCR (*n* = 2). Expression tended to increase at 0.25 and 1 nmol/L, although there was no statistically significant difference. (B and C) Dose dependency of BMP7‐induced cadherin‐11 protein. Cells were incubated with various concentrations of BMP7 and lysed for immunoblot analysis. A representative immunoblot (B) and quantitative analysis (C, *n* = 3). *P < 0.05 versus 0 nmol/L. (D and E) ERK and p38 mediate intermediate‐dose BMP7‐induced cadherin‐11 expression. Cells were incubated with BMP7 1 nmol/L with PD98059 (PD) 5 *μ*mol/L, SB203580 (SB) 5 *μ*mol/L, or both and lysed for immunoblot analysis. A representative immunoblot (D) and quantitative analysis (E, *n* = 3). VEH, vehicle; *P < 0.05 versus CON.

Cadherin 6 or E‐cadherin, assessed by qPCR and/or immunoblot, was not detected in basal state nor induced by any concentrations of BMP7 in MS7 (data not shown). The assays were validated with positive controls (fetal mouse kidney). On the other hand, CDH11 assessed by qPCR and immunoblot was present in MS7 and increased by incubation with intermediate‐dose BMP7 for 24 h (Fig. 4A–C). BMP7‐induced upregulation of CDH11 was not affected by SB203580 5 *μ*mol/L or PD98059 5 *μ*mol/L alone, but was reversed by cotreatment with both (Fig. 4D and E).

### Effect of siRNA for cadherin‐11 in organ culture

**Figure 5:**
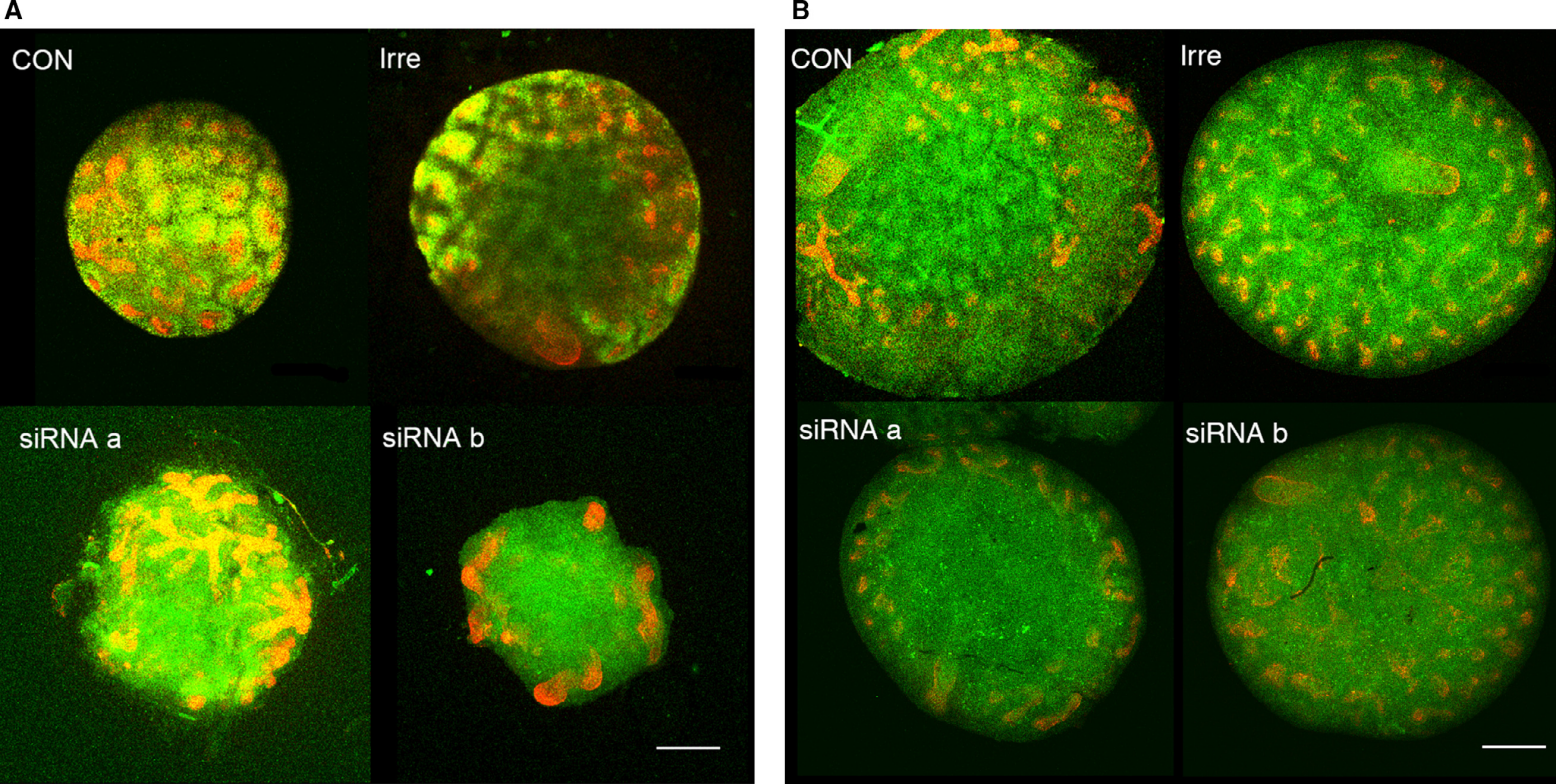
Effect of siRNA for cadherin‐11 in embryonic day 13 metanephroi. Embryonic day 13 metanephroi were transfected with irrelevant siRNA, or two different siRNA for CDH11 (a, b) for 24 h, then cultured with 10% DMEM for further 48 h. (A) Metanephroi were stained with anti‐Six2 antibody (green) and pancytokeratin (red). (B) Metanephroi were stained with anti‐CDH11 antibody (green) and pancytokeratin (red). CON, control with no siRNA; Irre, irrelevant siRNA. Scale bars, 250 *μ*m.

**Figure 6:**
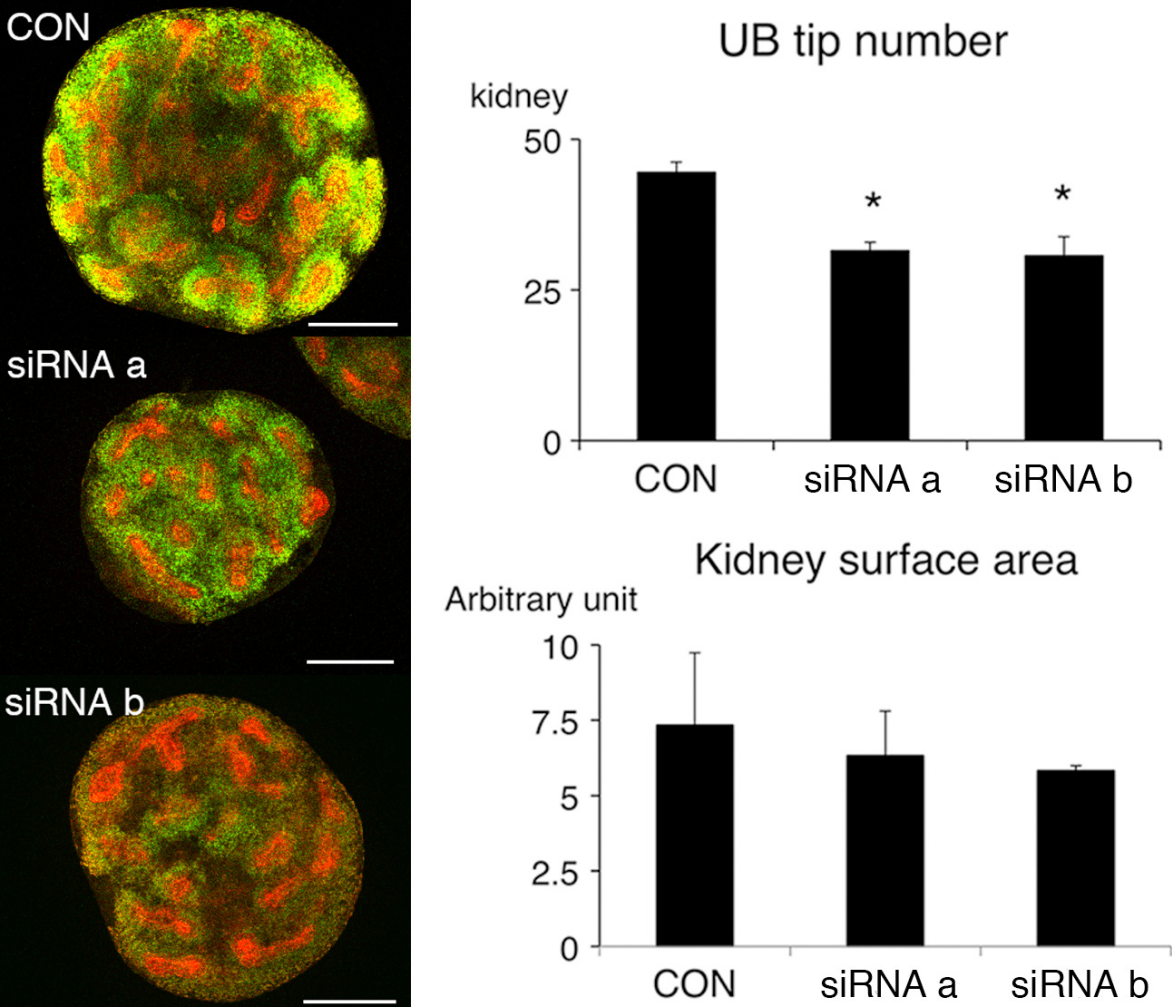
Effect of siRNA for cadherin‐11 in embryonic day 12 metanephroi. Embryonic day 12 metanephroi were transfected with irrelevant siRNA or two different siRNA for CDH11 (a, b) for 24 h, then cultured with 10% DMEM for further 24 h. Left column; metanephroi were stained with anti‐Six2 antibody (green) and pancytokeratin (red). Metanephroi cultured with irrelevant siRNA were similar to control (not shown). Right column: quantitative analysis of ureteric tip number and kidney surface area (*n* = 3). The number of ureteric bud tips was counted observing z‐stack images. Representative images were selected from one of z‐stack images. CON, control with no siRNA; *P < 0.05 versus CON. Scale bars, 250 *μ*m.

Embryonic day 13 mouse metanephroi were transfected with two different siRNAs for CDH11 and further cultured for 2 days. Cap mesenchyme marked by Six2 was well observed around ureteric tips in control metanephroi and those transfected with irrelevant siRNA (Fig. 5A). On the other hand, in metanephroi transfected with siRNAs, Six2‐positive cells were diffusely distributed and condensation around the ureteric tips was hardly observed. In metanephroi transfected with irrelevant siRNA, strong CDH11 expression was observed around ureteric tips (Fig. 5B). On the other hand, CDH11 expression was diffused and reduced in metanephroi transfected with siRNA a or b. To accurately count the number of ureteric bud tips, embryonic day 12 metanephroi were transfected and further cultured for 1 day (Fig. 6). The number of ureteric tips of transfected metanephroi was significantly less compared with those of nontransfected metanephroi. Kidney surface area of transfected metanephroi tended to be smaller, although the statistical significance was not achieved.

## Discussion

The present study showed that BMP7 increased proliferation and CDH11 expression in dose‐dependent fashion in a metanephric mesenchymal cell line. These effects of BMP7 on proliferation and CDH11 expression correspond to the differential activation of ERK and p38, and were suppressed by the inhibition of either or both, respectively.

We used the concentrations of BMP7, which previous studies have shown to be effective in collecting duct cells (Piscione et al. 1997, 2001; Hu et al. 2004). In the ureteric bud, which differentiates into the collecting duct, a concentration gradient of BMP7 is considered to exist with higher concentration at the tip and lower level in the trunk. The low (0.25 nmol/L) to high (28 nmol/L) concentrations were determined as those which exert effects on the proliferation of cultured collecting duct cells similar to those observed in tissue explants (Piscione et al. 2001).

The effect of BMP7 on metanephric mesenchyme has previously been investigated only in organ culture. Thus, 0.25 nmol/L BMP7 increased kidney surface area, while 1 nmol/L BMP7 exerted a negative effect on explant growth (Piscione et al. 1997). These results are in accord with the present study in which BMP7 exerts contradictory effects on proliferation depending on the concentration. In the stroma or cap mesenchyme distant from the tip, BMP7 concentration is considered to be lower, and metanephric mesenchymal cells proliferate. Near the tip of ureteric bud, on the other hand, the growth may be less or even inhibited and cells may be primed for differentiation. Recently, Tanigawa et al. (2016) reported that high concentrations of BMP7 (more than 1.6 nmol/L) reduced the percentage of nephron progenitor Six2‐positive cells, while lower concentrations (0.3 nmol/L) were optimal for the expansion of this cell population. Within cap mesenchyme also, therefore, there may be a gradient of proliferation depending on the distance from the ureteral tip.

In a similar manner to collecting duct cells and ureteric bud branching, the effects of BMP7 on cell signaling were dose dependent (Piscione et al. 1997; Hu et al. 2004). In murine inner medullary collecting duct (mIMCD) cells, BMP7 stimulated p38 but not ERK or JNK. In contrast to metanephric mesenchymal cells, p38 was stimulated and inhibited by low dose (0.25 nmol/L) and high dose (10 nmol/L) of BMP7, respectively, in mIMCD cells (Hu et al. 2004). Furthermore, the time course of cell signaling was different. In mIMCD cells, p38 was activated at 1 h after stimulation with BMP7. Most of the previous studies reporting the activation of MAP kinases, in fact, demonstrated stimulation at earlier time points. TAK1 or integrin‐linked kinase has been shown to mediate the rapid activation of p38 by BMP7 (Leung‐Hagesteijn et al. 2005). In the present study, both p38 and ERK were activated at 24 h, up to which point most previous studies had not examined the time course. Gai et al.'s (2009) study was in accord with our study, which reported activation of p38 at 48 h with approximately 1 nmol/L BMP7 in metanephric mesenchymal cells. Delayed activation of p38 via Smad‐dependent pathway by TGF‐β has been reported in pancreatic carcinoma cells (Takekawa et al. 2002). Similar mechanism may be operative for the BMP7‐induced late activation of p38 or ERK in metanephric mesenchymal cells.

While p38 is activated by higher concentration of BMP7 than ERK, SB203580 inhibited low‐dose BMP7‐induced proliferation in a similar manner to PD98059 suggesting that p38 is also involved in the signaling of low‐dose BMP7. In fact, time course study showed activation at 24 h by low‐dose BMP7 when compared with 0 h. We think that there was a slight activation of p38, which was not detectable by immunoblot and/or obscured by variability. A previous study reported that BMP7 promoted nephron progenitor cell proliferation through TAK1, JNK, and a transcription factor Jun (Muthukrishnan et al. 2015). In that study, the activation of JNK was observed at 15 min, and the concentration of BMP7 to stimulate proliferation and JNK was approximately 3 nmol/L. Whether ERK and p38 are also activated at later time points and involved in the proliferation of progenitor cells in a similar manner to metanephric mesenchymal cells remains unknown.

What are the roles of BMP7 around the tip of ureteric buds where the concentrations are thought to be higher? The present study showed that upregulation of CDH11 is one of them. The siRNA transfection studies demonstrate that CDH11 is necessary for cap mesenchyme formation. Cadherin 6 or cadherin E was not induced by BMP7 generally consistent with previous studies. Thus, WNT signal‐mediated differentiation was not induced by BMP7 even at high concentrations (Brown et al. 2013; Tanigawa et al. 2016). BMP7 only primes nephron progenitors to become responsive to WNT signaling toward epithelialization. Our findings, however, are inconsistent with those by Gai et al. (2009). They reported that BMP7 of a similar concentration to our intermediate‐dose BMP7 induced E‐cadherin expression through p38 in primary cultured metanephric mesenchymal cells. While the activation of p38 is in agreement with our study, CDH11 but not E‐cadherin was induced by BMP7 in MS7. Since they used primary metanephric mesenchymal cells isolated from rudiments of rat embryonic day 13.5, it is possible that the cells were contaminated by ureteric bud cells or contained glomerular epithelial cells. Alternatively, the discrepancy could be due to the difference between primary cells and immortalized cell line or the antibody used.

CDH11 has been shown to promote cell migration involved in mesenchymal condensation (Simonneau et al. 1995). In the development of heart and tendon, CDH11‐mediated cell condensation is a prerequisite. In the developing kidney, it is expressed in metanephric mesenchymal cells most strongly in those surrounding the ureteric bud tips (Challen et al. 2004). Recently, CDH11 has been proposed as a regulator of stem cell fate decisions (Alimperti and Andreadis 2015). More recently, CDH11 has been shown to localize adherens junctions, which is important in diverse functions of stem cell biology including the maintenance and differentiation (Langhe et al. 2016). In the present study, in the metanephroi cultured with siRNA for CDH11 for 3 days, cap mesenchyme was hardly observed. This is reminiscent of the metanephroi cultured with SB203580 in our previous study, in which WT1‐positive cells were distributed loosely in mesenchyme with slight signs of condensation around ureteric buds (Hida et al. 2002). Furthermore, SB203580‐treated metanephroi were small with reduced glomerular number. The effect of PD98059 is less severe, and the metanephros size was maintained and cap mesenchyme, vesicles, comma‐, and S‐shaped bodies were observed, although the number was reduced. These phenotypes may be due at least in part to the inhibited signaling of BMP7 via p38 or ERK. BMP7 deletion results in hypocellular kidneys with few nephrons, apoptosis, and accumulation of loose interstitial mesenchyme similar to the kidneys treated with SB203580 (Dudley et al. 1995).

Culturing earlier stage metanephroi for 2 days allowed the measurement of ureteric bud tip number, which was reduced by siRNA for CDH11. This is considered to be due to the inhibited signals from cap mesenchyme to enhance ureteric branching. Decreased metanephros size may be due to inhibited CDH11 in the stroma and/or reduced signal from the ureteric bud.

Of interest, CDH11 has been reported to be upregulated in the embryonic kidney of offspring of rats on low‐protein diet and rats treated with hydrocortisone (Welham et al. 2005; Chan et al. 2010). We have also observed that maternal nutrient restriction reduced and increased the expression of CDH11 in the embryonic days 15 and 18 kidney, respectively (M. Awazu, unpubl. obs.). These kidneys are characterized by reduced nephron number, and CDH11 may be mediating, at least in part, the effect of adverse intrauterine environment.

The effect of inhibiting ERK or p38 pathway on proliferation and CDH11 upregulation was different. Thus, proliferation was inhibited by either PD98059 or SB203580 alone, whereas CDH11 expression was inhibited by the coincubation with both. In other words, combined activation of ERK and p38 is needed for cell proliferation, and isolated activation of either suffices for CDH11 upregulation. While JNK, Smad, and integrin kinase other than p38 and ERK have been shown to be involved in proliferative actions of BMP7 (Leung‐Hagesteijn et al. 2005; Blank et al. 2009), not much is known regarding the regulation of CDH11. CDH11 has been reported to be upregulated by TGF‐β1 via ERK in cardiac myofibroblasts (Wang et al. 2014). A transcription factor COUP‐TFII is reported to upregulate CDH11 expression in a carcinoma cell line and speculated to play a role in the cadherin‐11 to cadherin‐6 switch during kidney development (Bringuier et al. 2015). Regulatory sequence of CDH11, however, has not been defined.

Smad proteins are the downstream signaling molecules of BMP7 and have been shown to function in collecting duct cells and nephron progenitor cells (Piscione et al. 2001; Brown et al. 2013). They may also be involved in BMP7‐induced proliferation and CDH11 expression in metanephric mesenchymal cells. All Smads (the receptor‐regulated Smad1, 2, 3, 5, 8, the common partner Smad4, and inhibitory Smad6, 7) are expressed by mesenchymal cells in the nephrogenic zone (Simic and Vukicevic 2005). Of interest, Oxburgh et al. (2004) reported that Smad4 null mesenchyme cells do not aggregate tightly around the ureteric bud tips in a similar manner to metanephroi treated with siCDH11. Future studies are needed to explore the role of Smads as a downstream signaling molecule of BMP7 in metanephric mesenchymal cells.

**Figure 7:**
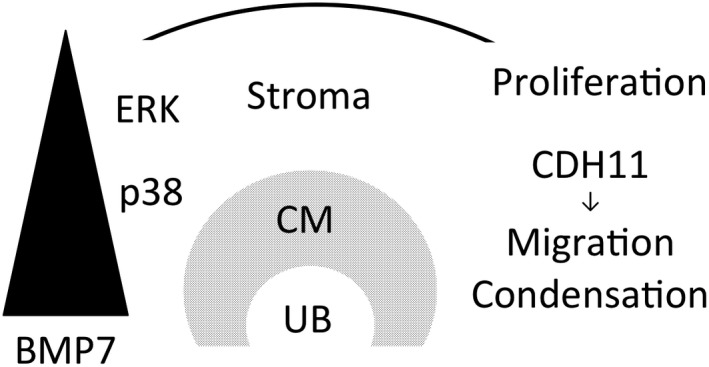
Hypothetical action of BMP7 in metanephric mesenchyme. A concentration gradient of BMP7 is considered to exist, being higher around the tip of ureteric bud (UB) and cap mesenchyme (CM) and becoming lower toward the periphery. ERK and p38 are activated at low and intermediate concentration of BMP7, respectively. BMP7 stimulates proliferation at low to intermediate concentration, whereas induces cadherin‐11 (CDH11) expression at intermediate concentration. Both effects are mediated by ERK and p38. BMP7 thus maintains metanephric mesenchymal cells in the stroma and stimulates migration and condensation via CDH11 in cap mesenchyme.

In conclusion, we reported the differential functional roles of BMP7 in metanephric mesenchymal cells, proliferation, and upregulation of CDH11. Its concentration‐dependent function is summarized in Figure 7, which supports the classic hypothesis that BMP7 acts as a morphogen.

## Conflict of Interest

There is no conflict of interest to disclose.
